# Comparison of Microbial Community and Metabolites in Four Stomach Compartments of Myostatin-Gene-Edited and Non-edited Cattle

**DOI:** 10.3389/fmicb.2022.844962

**Published:** 2022-03-24

**Authors:** Xinyu Zhou, Mingjuan Gu, Lin Zhu, Di Wu, Miaomiao Yang, Yajie Gao, Xueqiao Wang, Chunling Bai, Zhuying Wei, Lei Yang, Guangpeng Li

**Affiliations:** ^1^State Key Laboratory of Reproductive Regulation and Breeding of Grassland Livestock, Inner Mongolia University, Hohhot, China; ^2^School of Life Sciences, Inner Mongolia University, Hohhot, China

**Keywords:** myostatin, stomach, microbiome, metabolome, cattle, gene edit

## Abstract

Myostatin (MSTN), a major negative regulator of skeletal muscle mass and an endocrine factor, can regulate the metabolism of various organisms. Inhibition of the *MSTN* gene can improve meat production from livestock. Rumen microorganisms are associated with production and health traits of cattle, but changes in the microbial composition and metabolome in the four stomach compartments of *MSTN* gene–edited cattle have not previously been studied. Our results indicated that microbial diversity and dominant bacteria in the four stomach compartments were very similar between *MSTN* gene–edited and wild-type (WT) cattle. The microbiota composition was significantly different between *MSTN* gene–edited and WT cattle. Our results show that the relative abundance of the phylum Proteobacteria in the reticulum of *MSTN* gene–edited cattle was lower than that of WT cattle, whereas the relative abundance of the genus *Prevotella* in the omasum of *MSTN* gene–edited cattle was significantly higher than that of WT cattle. Metabolomics analysis revealed that the intensity of L-proline and acetic acid was significantly different in the rumen, reticulum, and abomasum between the two types of cattle. Meanwhile, pathway topology analysis indicated that the differential metabolites were predominantly involved in arginine biosynthesis and glutamate metabolism in the rumen, reticulum, and omasum but were mainly involved in pyruvate metabolism and glycolysis/gluconeogenesis in the abomasum. Spearman correlation network analysis further demonstrated that there was a significant correlation between microflora composition and metabolic pathways. These findings provide clues for studying nutrient digestion and absorption ability of *MSTN* gene–edited cattle.

## Introduction

Myostatin (MSTN), also known as growth and differentiation factor 8, is a major regulator of skeletal muscle development (Beyer et al., 2013). Inhibition of the *MSTN* gene can improve the meat production performance of livestock, and gene-editing technology provides the possibility to obtain animals with double-muscle traits (Lee et al., 2017). A visibly distinct muscular hypertrophy, commonly known as double muscling (Kambadur et al., 1997). Researchers have successfully obtained *MSTN*-mutant sheep, cattle, pigs, and goats by gene-editing technology (Proudfoot et al., 2015; Wang et al., 2015; Yu et al., 2016). Our research group has also successfully obtained MSTN gene–edited cattle (Gao et al., 2020). Physiologically, the myostatin gene not only suppresses skeletal muscle growth (Deng et al., 2017) but also impacts metabolic function (Huang et al., 2011), affecting both glucose and fat metabolism (Elliott et al., 2012).

Gut microbiota perform different functions in the animals, including metabolic and protective structure (Chiofalo et al., 2004), and improve performance (Angelakis, 2017). In addition, gut microbiota regulated various metabolic processes in the host, including energy homeostasis, glucose metabolism, and lipid metabolism (Sonnenburg and Bäckhed, 2016). The gut microbiota was important for host physiology, homeostasis, and sustained health and also affect skeletal muscle mass in mice, suggesting the existence of a gut microbiota-skeletal muscle axis (Lahiri et al., 2019). Recent studies reported that host genetics also influence gut microbial composition (Goodrich et al., 2016; Turpin et al., 2016; Li et al., 2019). In *MSTN*-mutated pigs, analyses of the microbiome and metabolome of jejunum and cecum contents indicated that the composition of metabolites and microbial strains were significantly different from those of wild-type (WT) pigs (Pei et al., 2021). The microbial strains in the rectum of *MSTN^–/–^* pigs were also different to those of WT pigs, but there were no adverse effects on fecal microbiota compositional structure in the *MSTN^–/–^* pigs compared with the WT pigs (Cui et al., 2019). These findings suggest that mutation of the gene *MSTN* in monogastric pigs induces changes in intestinal metabolites and some microorganisms. In ruminants, the four stomach compartments—rumen, reticulum, omasum, and abomasum—have different physiological functions (Xin et al., 2019; Chong et al., 2020). Food initially flows through the rumen and reticulum, then passes to the omasum, and finally into the abomasum. The rumen, reticulum, and omasum contain numerous microorganisms that contribute to anaerobic degradation of nutrients (Yu L.et al., 2018). Studies have shown that natural or artificial mutations of *MSTN* significantly improve skeletal muscle development (Gao et al., 2020; Lee et al., 2020); however, it is not clear whether *MSTN* mutation in cattle induces changes in the microbiota of the four stomach compartments.

In the present study, metagenome sequencing and metabolome were employed to investigate changes in microbial composition and metabolites in the rumen, reticulum, omasum, and abomasum of *MSTN* gene–edited cattle (*MSTN^+/–^ cattle*) compared with WT cattle.

## Results

### Diversity Analyses of Microbiota in the Rumen, Reticulum, Omasum, and Abomasum of *MSTN*^+/–^ and Wild-Type Cattle

A total of 1,872,117,574 reads were generated by metagenome sequencing, and 1,761,690,042 clean reads were retained after quality control and host gene removal. *De novo* assembly resulted in 4,232,512 contigs, and the grand total of N50 length was 28,246 base pairs (Supplementary Table 1).

**FIGURE 1:**
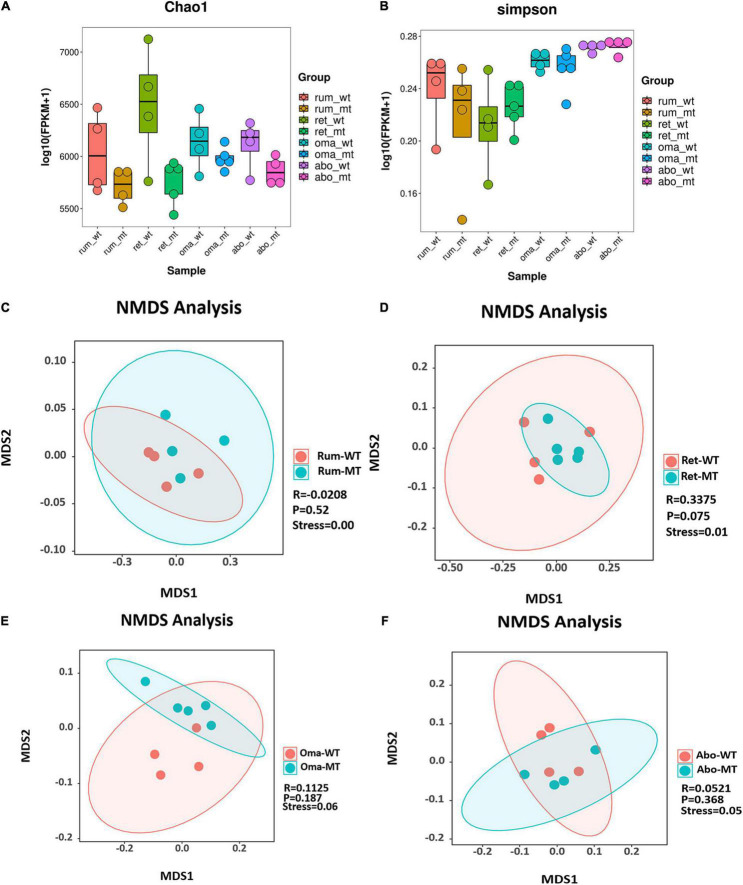
Diversity and structural analysis of *MSTN*^+/–^ and WT cattle. Box plots showing chao1 **(A)** and Simpson **(B)** of indices in both *MSTN*^+/–^ and WT four stomach samples. Non-metric multidimensional scaling (NMDS) ordination plot assessed by Bray–Curtis in both *MSTN*^+/–^ and WT rumen **(C)**, reticulum **(D)**, omasum **(E)**, and abomasum samples **(F)**. Stress values for ordination plot were < 0.1, which indicated the accuracy of data representation in a two-dimensional space. When stress values < 0.05, it has a good representative. rum, rumen; ret, reticulum; oma, omasum; abo, abomasum; WT, wild-type cattle; MT, MSTN^+/–^ cattle.

Comparison of differences in stomach microbes and bacterial diversity using Chao1 and Simpson indices of microbial richness indicated that there were no statistical differences among the four stomach compartments between *MSTN*^+/–^ and WT cattle (Figures 1A,B). Furthermore, no differences were observed in Shannon and observed species between *MSTN*^+/–^ and WT cattle (Supplementary Figures 1A,B). β-Diversity analysis allowed non-linear relationships between samples to be revealed. On the basis of the accurate NMDS maps and data in a two-dimensional space, the β-diversity of the rumen and reticulum had a good representation (stress value < 0.05) (Figures 1C,D). The NMDS-based maps of the omasum and abomasum also showed a good ranking (stress value < 0.1) (Figures 1E,F). These findings indicated that the *MSTN* mutation did not have a significant effect on bacterial diversities and richness, but the species composition had changed between the *MSTN*^+/–^ cattle and the WT cattle.

### Bacterial Composition in the Rumen, Reticulum, Omasum, and Abomasum of *MSTN*^+/–^ and Wild-Type Cattle

**FIGURE 2:**
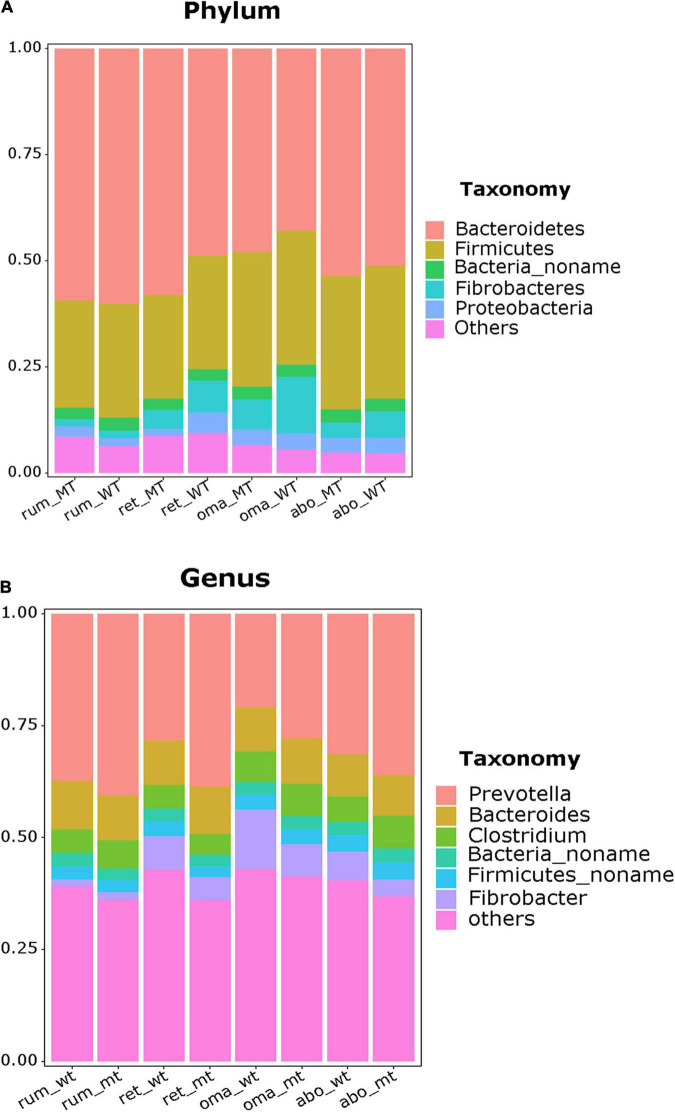
Compositional profiles of the stomach microbiome between *MSTN*^+/–^ and WT cattle. Bacterial composition at the phylum **(A)** and genus levels **(B)** in the rumen, reticulum, omasum, and abomasum.

In this section, the relative abundance at the phylum and genus levels in the four stomach compartments were compared using rank sum test, and the *p*-values were also be shown (Supplementary Table 2). In the rumen, the phylum Bacteroidetes were the dominant bacteria (*MSTN*^+/–^ = 56.4% relative abundance, *WT* = 60.2%), followed by Firmicutes (*MSTN*^+/–^= 26.6%, *WT* = 26.5%), with no differences in relative abundance of these taxa between *MSTN*^+/–^ and WT cattle (Figure 2A). At the genus level, the predominant bacteria were *Prevotella* (*MSTN*^+/–^ = 38.1%, *WT* = 37.7%), followed by *Bacteroides* (*MSTN*^+/–^ = 9.7%, *WT* = 10.8%) and *Clostridium* (*MSTN*^+/–^ = 10.6%, *WT* = 9.4%), with no differences in relative abundances of these taxa between *MSTN*^+/–^ and WT cattle (Figure 2B).

Similarly, in the reticulum, the phylum Bacteroidetes (*MSTN*^+/–^ = 58.2%, *WT* = 47.9%) were the dominant taxa, followed by Firmicutes (*MSTN*^+/–^ = 24.3%, *WT* = 26.9%), with no differences in relative abundances of these taxa between *MSTN*^+/–^ and WT cattle. However, the relative abundance of the phylum Proteobacteria (*MSTN*^+/–^ = 1.72%, *WT* = 4.91%, *p* = 0.0093) was significantly lower than that in WT cattle (Figure 2A). At the genus level, the paramount bacteria were also *Prevotella* (*MSTN*^+/–^ = 38.8%, *WT* = 27.5%), followed by *Bacteroides* (*MSTN*^+/–^ = 10.5%, *WT* = 9.7%) and *Clostridium* (MSTN^+/–^ = 5.2%, *WT* = 9.5%), with no differences in relative abundances of these taxa between *MSTN*^+/–^ and WT cattle (Figure 2B).

In the omasum, the predominant bacterial phyla were Bacteroidetes (*MSTN*^+/–^ = 42.9%, *WT* = 47.9%) and Firmicutes (*MSTN*^+/–^ = 31.5%, *WT* = 31.6%), with no difference in relative abundance of these taxa between *MSTN*^+/–^ and WT cattle (Figure 2A). At the genus level, *Prevotella* was the prevalent bacterial taxa in both *MSTN*^+/–^ and WT cattle, but the relative abundance of *Prevotella* in *MSTN*^+/–^ cattle (27.89%) was significantly higher than that in WT cattle (20.80%) (*p* = 0.03). The second most abundant genus in the omasum was *Fibrobacter* for WT cattle and *Bacteroides* for *MSTN*^+/–^ cattle (Figure 2B).

For the abomasum, Bacteroidetes was the dominant phylum (*MSTN*^+/–^ = 53.7%, *WT* = 51.1%), followed by Firmicutes (*MSTN*^+/–^ = 31.3%, *WT* = 31.4%), with no difference in relative abundances of these two taxa between *MSTN*^+/–^ and WT cattle (Figure 2A). The genera *Prevotella* (*MSTN*^+/–^ = 36.1%, *WT* = 31.4%) and *Bacteroides* (*MSTN*^+/–^ = 9.0%, *WT* = 9.4%) were the predominant bacteria in both *MSTN*^+/–^ and WT cattle (Figure 2B). However, the third most abundant genus differed between the two types of cattle; *Clostridium* and *Fibrobacter* were the third most abundant genus in *MSTN*^+/–^ cattle and WT cattle, respectively. Although the *MSTN* gene mutation had no effect on the abundance of dominant microbiota, bacterial composition at the genus level was changed in both the omasum and the abomasum of *MSTN*^+/–^ cattle compared with WT cattle.

### Metabolome Analysis in the Rumen, Reticulum, Omasum, and Abomasum of *MSTN*^+/–^ and Wild-Type Cattle

**FIGURE 3:**
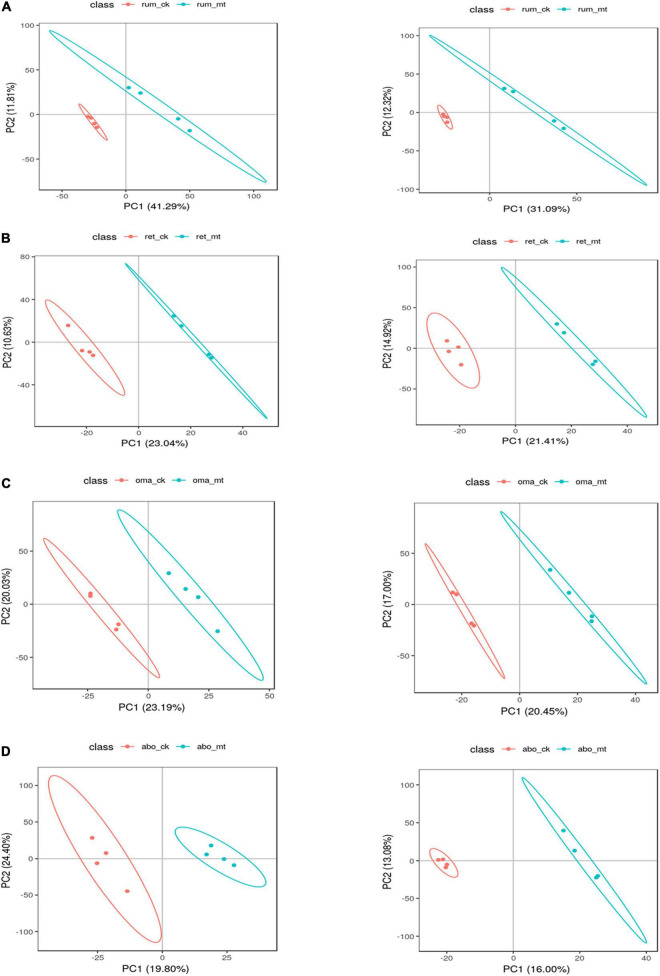
Differential metabolites analysis of the stomach between the *MSTN*^+/–^ and WT cattle. PLS-DA plot of fecal metabolites detected in negative (left) and positive (right) mode of *MSTN*^+/–^ and WT cattle in the rumen **(A)**, reticulum **(B)**, omasum **(C)**, and abomasum **(D)**.

After *t*-test and VIP (variable importance in projection) filtering, the number of the differential metabolites in the rumen, reticulum, omasum, and abomasum between *MSTN*^+/–^ and WT cattle was 51, 45, 31, and 34, respectively. For further analysis, partial least-squares discriminant analysis (PLS-DA) was used to identify metabolite changes in the four stomach compartments of *MSTN*^+/–^ and WT cattle. This analysis showed significant differences between *MSTN*^+/–^ and WT samples in the same stomach compartments (Figures 3A–D). HMDB (Human Metabolome Database) was then used to categorize the differential metabolites according to the properties of the compounds (Supplementary Tables 3–6). Lipids and lipid-like molecules and organic acids and their derivatives accounted for a large proportion of the metabolites in all four stomach compartments (Supplementary Figure 2). This suggested that the *MSTN* gene mutation has a marked effect on amino acid and lipid metabolism in the four stomach compartments of cattle.

### Core Microbiota and Different Metabolites Throughout the Rumen, Reticulum, Omasum, and Abomasum in *MSTN*^+/–^ and Wild-Type Cattle

**FIGURE 4:**
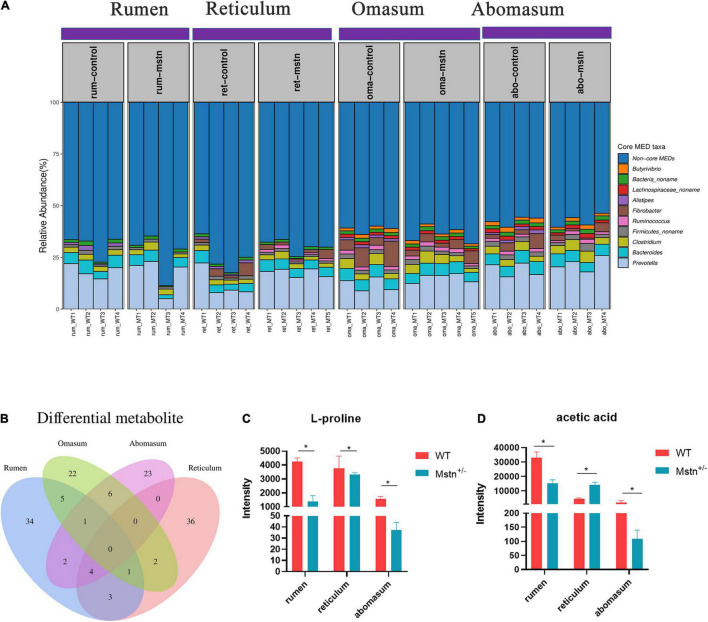
The core microbiota and different metabolite throughout the rumen, reticulum, omasum, and abomasum between the *MSTN*^+/–^ and WT cattle. Relative abundances of minimum entropy decomposition (MED) nodes identified as core members (taxa present at greater than 1% abundance in more than 50% of samples) **(A)**. The Venn diagram of the shared and unique metabolites throughout the four stomach compartments **(B)**. L-proline **(C)** and acetic acid **(D)** intensity between *MSTN*^+/–^ and WT cattle in the rumen, reticulum, and abomasum. *p < 0.05.

Relative abundance of minimum entropy decomposition (MED) nodes was used to identify core members of the microbiota in the four compartments of the ruminants’ stomachs. There were sharp shifts in locations of core bacterial compositions throughout the four stomach compartments, which was mainly driven by 10 distinct MEDs (MED nodes with relative abundances > 1% in > 50% of samples in each stomach location). The abundance of the 10 cores microbiota in the ruminant stomach (the four compartments) was no difference between MSTN and WT cattle. Five of the 10 core bacterial groups—*Prevotella*, *Bacteroides*, *Clostridium*, *Firmicutes_noname*, and *Bacteria_nonam*e—were common to all four stomach chambers. The relative abundance of these five bacterial throughout the four compartments had no difference in WT cattle. The relative of *Clostridium* in abomasum was higher than rumen and reticulum, and *Bacteria_noname* in rumen was lower than reticulum and omasum of *MSTN*^+/–^ cattle. However, the *Butyrivibrio* and *Lachnospiraceae_noname* were only in the omasum and abomasum regarded as core bacteria (Figure 4A). Venn diagrams of differential metabolites showed that no common metabolites were shared by the four stomach compartments. However, four metabolites were shared by the rumen, reticulum, and abomasum; these metabolites were 4-acetyl-2(3H)-benzoxazolone, 1H-indole-3-carboxylic acid, L-proline, and acetic acid. The intensity of L-Proline was lower in *MSTN*^+/–^ cattle compared with that of WT cattle, and acetic acid exhibited the same trend except in the reticulum (Figures 4B–D). These results indicated that sharp shifts in locations of core microbiota have existed, but the *MSTN* mutation had no effect on the changes. Although the number of the differential metabolites in the four stomach compartments was different, four differential metabolites were changed in rumen, omasum, and abomasum.

### Pathway Topology Analysis in the Rumen, Reticulum, Omasum, and Abomasum of *MSTN*^+/–^ and Wild-Type Cattle

**FIGURE 5:**
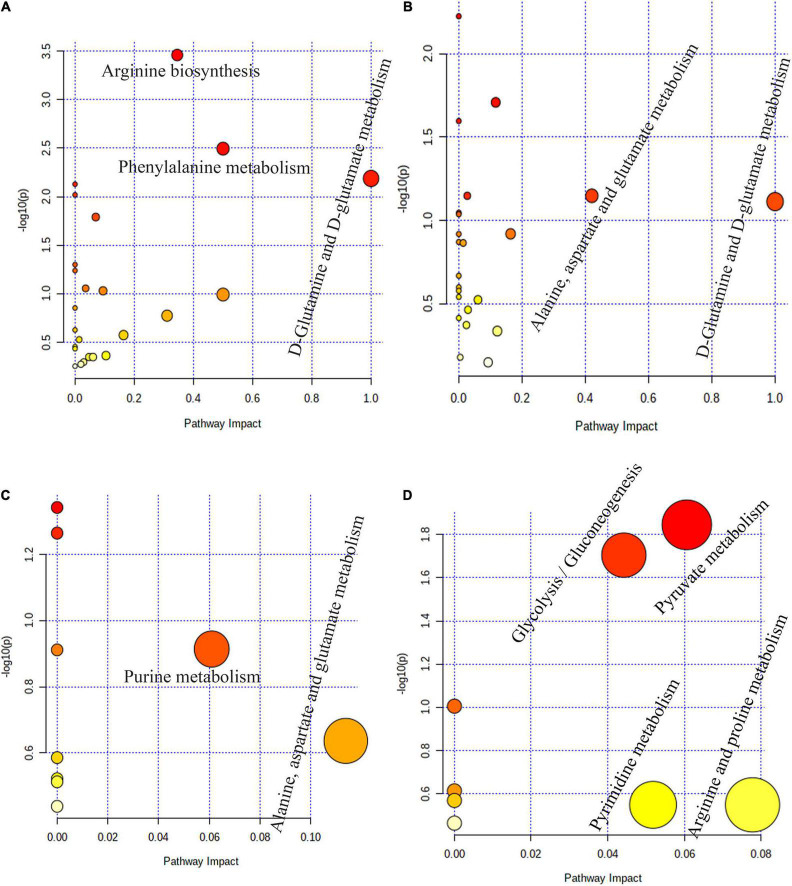
Pathway enrichment analysis based on stomach different metabolites. Pathway enrichment analysis performed using the significantly different metabolites between *MSTN*^+/–^ and WT cattle in rumen **(A)**, reticulum **(B)**, omasum **(C)** and abomasum **(D)**.

Pathway topology analysis based on significantly different metabolites in the four stomach compartments of *MSTN*^+/–^ and WT cattle revealed the pathways that were most impacted by the *MSTN* mutation. Three main metabolic pathways of “D-glutamine and D-glutamate metabolism,” “phenylalanine metabolism,” and “arginine biosynthesis” were observed in the rumen (Figure 5A). In the reticulum, two main metabolic pathways were detected, “D-glutamine and D-glutamate metabolism” and “alanine, aspartate, and glutamate metabolism” (Figure 5B). Two main metabolic pathways of “alanine, aspartate, and glutamate metabolism” and “purine metabolism” were observed in the omasum (Figure 5C), whereas four main metabolic pathways, comprising “arginine and proline metabolism,” “pyruvate metabolism,” “pyrimidine metabolism,” and “glycolysis/gluconeogenesis,” were detected in the abomasum (Figure 5D). Collectively, these results indicated that *MSTN* gene mutation significantly influenced amino acid metabolism and energy metabolism in four stomach compartments of the cattle.

### Correlation of Differential Microbiota With Enrichment Pathways

**FIGURE 6:**
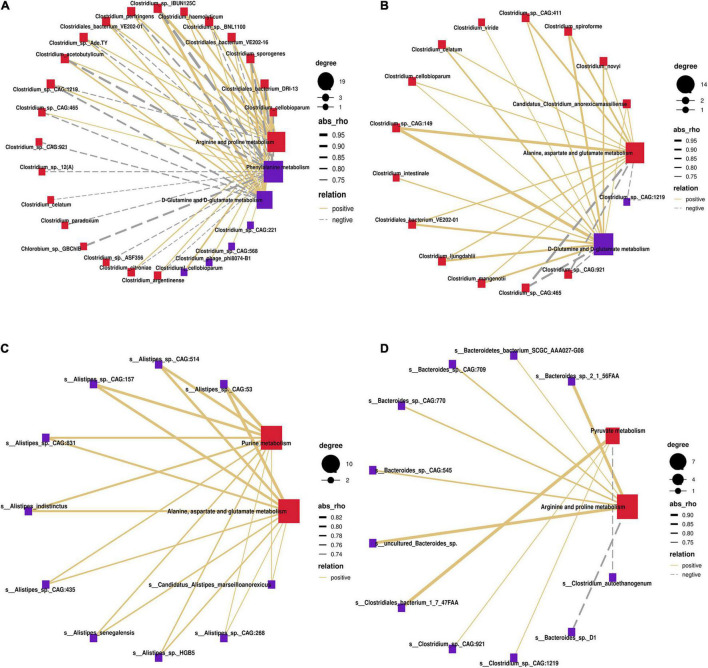
Correlation of differential bacteria at the species level with enrichment pathway. Spearman’s correlation network showing relationships between microbiota and different metabolites enrichment pathways in rumen **(A)**, reticulum **(B)**, omasum **(C)**, and abomasum **(D)**. Only partly strong correlations (*R* > 0.7 or *R* < −0.7, *P* < 0.05) were showed in the correlation networks. The solid and dashed lines indicate positive and negative correlations, respectively.

To identify potential stomach microbiome and host metabolic interactions that may differ between MSTN and WT cattle, Spearman hierarchical correlation networks between differential species and the changed metabolism pathways in the four stomach compartments were created, respectively. In the rumen, species of the genus *Clostridium* accounted for the largest proportion of the correlation networks. *Clostridium*_sp._IBUN125C exhibited the strongest positive correlation with “arginine and proline metabolism” and “D-glutamine and D-glutamate metabolism” but showed the strongest negative correlation with “phenylalanine metabolism” (Figure 6A). Species of the genus *Clostridium* also occupied the largest proportion of the correlation networks in the reticulum. *Clostridium*_sp._CAG:149 showed the strongest positive correlation with “D-glutamine and D-glutamate metabolism” and “alanine, aspartate, and glutamate metabolism” (Figure 6B). The omasum was different from the rumen and the reticulum, with species of *Alistipes* comprising the largest proportion of the correlation networks. All species of the genus *Alistipes* (especially *Alistipes*_sp._CAG:53) were positively correlated with “alanine, aspartate, and glutamate metabolism” and “purine metabolism” (Figure 6C). In the abomasum, species of the genera *Bacteroides* and *Clostridium* accounted for the highest proportion of the correlation networks. Species of the genus *Bacteroides* were mainly significantly related to “arginine and proline metabolism,” whereas species of the genus *Clostridium* were significantly related to “pyruvate metabolism” (Figure 6D). Therefore, species of the genera *Clostridium*, *Alistipes*, and *Bacteroides* were strongly associated with the metabolic pathways affected by *MSTN* mutation.

## Discussion

Determination of microbial composition in the stomach compartments of ruminants is not only helpful to understand the physiological function of ruminants but also facilitates further development of animal husbandry (Peng et al., 2015). In the present study, an integrated approach of metagenomic sequencing and liquid chromatography-mass spectrometry (LC-MS)–based untargeted metabolomics was applied to examine the microbiota and metabolites in the four stomach compartments of cattle and determine the effect of *MSTN* gene editing on the microbiome and metabolome in these stomach compartments.

There were no significant differences in α-diversity of microbiota between *MSTN*^+/–^ and WT cattle. However, β-diversities in the four stomach compartments were markedly altered between *MSTN*^+/–^ cattle and WT cattle, suggesting that the microbiota composition in *MSTN*^+/–^ cattle was different from that of WT cattle. These results are consistent with a previous report on jejunum and cecum feces in *MSTN*-edited pigs (Pei et al., 2021). Firmicutes and Bacteroidetes were the most abundant bacterial phyla in rumen and reticulum liquid fractions and the rumen solid fraction during fattening of Japanese black beef cattle (Ogata et al., 2020). Furthermore, Xin et al. (2019) compared the microbiota in the rumen, reticulum, omasum, and abomasum of dairy, beef, and yak cattle and found that Firmicutes and Bacteroidetes were the abundant phyla in all four stomach compartments. Data from the current study indicate that Firmicutes and Bacteroidetes are also the most abundant phyla in *MSTN*^+/–^ cattle. In addition, there were no differences in the abundances of Firmicutes and Bacteroidetes between *MSTN*^+/–^ cattle and WT cattle. Consistent with these findings, Cui et al. (2019) reported that no significant differences were observed in Firmicutes and Bacteroidetes abundance in rectal feces between *MSTN^–/–^* and WT pigs. At the genus level, *Prevotella* was the predominant bacterial taxa in the four stomach compartments of both *MSTN*^+/–^ cattle and WT cattle. This is in accordance with some studies describing *Prevotella* as the most abundant genus in the rumen (Mao et al., 2015; Henderson et al., 2016; Pitta et al., 2016; Thomas et al., 2017). Although the dominant bacteria were similar in the omasum, the current study indicated a higher relative abundance of *Prevotella* in *MSTN*^+/–^ cattle compared with WT cattle. *Prevotella* play a major role in carbohydrate and nitrogen metabolism (Kim et al., 2017) and also function in the improvement of glucose metabolism in barley kernel–based bread (Kovatcheva-Datchary et al., 2015). In previous studies, *MSTN* mutation changed lipid metabolism and enhanced glycogenolysis and glycolysis in muscle tissue of *MSTN* gene–edited cattle (Yang et al., 2018; Xin et al., 2020). Therefore, *Prevotella* may be involved in muscle metabolism. It is concluded that *MSTN* gene mutation changes metabolism in the stomach compartments of ruminants and may be associated with *Prevotella*.

At the genus level, the current study also demonstrated that the 10 distinct MEDs exhibited significant location shifts throughout the four stomach compartments. *Ruminococcus*, *Fibrobacter*, *Butyrivibrio*, and *Lachnospiraceae* were the core MEDs with active location changes throughout the four stomach compartments, whereas *Prevotella* and *Bacteroides* were relatively stable throughout the four compartments. Rumen microbial fermentation provides ruminants with volatile fatty acids and microbial proteins. The reticulum, omasum, and abomasum are the iconic digestive organs of ruminants and are important colonization sites of many commensal microorganisms (Xue et al., 2018). *Ruminococcus*, *Fibrobacter*, and *Butyrivibrio* are involved in carbohydrate fermentation in the stomachs of ruminants (Ze et al., 2012; Neumann et al., 2017; Neumann and Suen, 2018; Palevich et al., 2019a,b). The movement of these bacteria in the four stomach compartments may be closely related to changes of metabolites in the compartments. Furthermore, we also comprehensively analyzed the metabolome in the four stomach compartments of these cattle. The metabolites, especially L-proline and acetic acid, in the four stomach compartments were significantly altered following *MTSN* mutation. Acetic acid is one of the short-chain fatty acids (SCFAs) produced by bacterial fermentation of non-digestible carbohydrate (den Besten et al., 2013). SCFAs influence lipid, carbohydrate, and protein metabolism in skeletal muscles, and acetic acid is considered the main SCFA metabolized by skeletal muscle tissue (Frampton et al., 2020). Therefore, the acetic acid changes in the stomach compartments suggest that MSTN may regulate SCFA metabolism. Functional analysis showed that differential metabolites were involved in amino acids metabolism (rumen, reticulum, and omasum), and energy metabolism (abomasum). In addition, pathway topology analysis indicated that arginine biosynthesis and glutamate metabolism were significantly affected by *MSTN* mutation. Arginine is not only a common amino acid in protein synthesis; together with glutamate, arginine can also be a sole source of nitrogen for *Escherichia coli* and a source of nitrogen, carbon, and energy for many other bacteria (Charlier and Bervoets, 2019). Considering the role of these pathways in microbial metabolism, we speculate that nitrogen metabolism in the rumen, reticulum, and omasum may be markedly changed in *MSTN*^+/–^ cattle.

Through network correlation analysis, species of the genera *Alistipes*, *Clostridium*, and *Bacteroides* were found to exhibit strong correlation with metabolic pathways that were influenced by *MSTN* mutation. A recent study reported that *Alistipes* regulated cholesterol homeostasis in the host (Le Roy et al., 2019), whereas a separate study found Clostridium was mostly devoted to acid production by degrading sugars, alcohols, amino acids, purines, pyrimidines, and polymers (Durre, 2014). Furthermore, significant correlations exist between *Bacteroides* and amino acids such as glutamine, glycine, and lysine (Oldiges et al., 2014). Both glycine and lysine stimulate protein synthesis and are regulated by AMP-activated protein kinase (AMPK) and mammalian target of rapamycin (mTOR) signaling pathways (Chen et al., 2020). However, MSTN may also regulate muscle mass development through the mTOR pathway (Xin et al., 2020). Therefore, these changes may be consistent with the double-muscle phenotype.

In conclusion, this study is the first to systematically compare the microbiome and metabolome of the four stomach compartments between *MSTN*^+/–^ cattle and WT cattle. Microbial α-diversity and dominant bacteria in the four stomach compartments were very similar. However, the microbiota composition in the reticulum and omasum were changed in the *MSTN*^+/–^ cattle compared with the WT cattle. The *MSTN* gene mutation significantly altered the metabolites of arginine biosynthesis and glutamate metabolism in the rumen, reticulum, and omasum, whereas the metabolites associated with pyruvate metabolism and glycolysis/gluconeogenesis in the abomasum were markedly changed. In conclusion, the *MSTN* gene mutation had a small effect on dominant microbiota but a large effect on amino acid metabolism in the four stomach compartments, which may help provide more useful nutrients for *MSTN* gene–edited cattle.

## Materials and Methods

### Animal Management

All animal procedures were reviewed and approved by the Committee on the Ethics of Animal Experiments at Inner Mongolia University. In this study, the hybrid *MSTN*^+/–^ cattle were produced by artificial insemination of the WT cows with the semen from the *MSTN* edited bull (Gao et al., 2020). The control cattle were WT Luxi cows mated with normal Luxi bull. Five 18-month-old *MSTN*^+/–^ bulls and four similar age WT bulls were raised under the same conditions at the Inner Mongolia University Beef Cattle Breeding Centre, Hohhot. The concentrate:roughage ratios were 25:75. The concentrate contained corn, barley, bran, and soybean cake. Nutrient component included saline, amino acids, crude protein, crude fiber, calcium, phosphorus, and trace elements. Roughage contained silage, hay, and alfalfa.

### Sample Collection

Before slaughter, the cattle were fasted for 24 h. The contents of the four stomachs were, respectively, collected from both *MSTN*^+/–^ and WT cattle, placed the samples into cryopreserved tubes, immediately put into liquid nitrogen, and stored at −80°C until use (Xin et al., 2019).

### Metagenome Sequencing and Analysis

DNA from different samples was extracted using the Stool DNA Kit (D4015-02, Omega, United States) according to the manufacturer’s instruction. The reagent that was designed to uncover DNA from trace amounts of samples has been proved effective for the preparation of DNA of most bacteria (Homer et al., 2008). Total DNA were eluted in 50 μl of elution buffer by a modification as described by the manufacturer (#69506, QIAGEN, Germany), and the samples were stored at −80°C until measurement in PCR (LC-BIO Technology, China). DNA library was constructed by the TruSeq Nano DNA LT Library Preparation Kit (FC-121-4001, Illumina, United States). In the end, metagenomic sequencing was performed using HiSeq4000 strategies. In addition, the sequencing cycles were eight with paired end.

Raw sequencing reads were processed to obtain valid reads for further analysis. Using the software Cutadapt (Version 1.9) (Martin, 2011), Fqtrim (Version 0.9.4), and Bowtie2 (Version 2.3.5.1) (Langmead and Salzberg, 2012) to remove the sequencing adapters, low-quality reads were trimmed and host contaminations were removed, respectively. After the quality-filtered reads were obtained, these were *de novo*–assembled to construct the metagenome for each sample by IDBA-UD v1.1.1 (Peng et al., 2012). All coding regions (CDS) of metagenomic contigs were predicted by MetaGeneMark v3.26 (Zhu et al., 2010). Next, CDS sequences of all samples were clustered by CD-HIT v4.6.1 (Li and Godzik, 2006) to obtain unigenes. The lowest common ancestor taxonomy of unigenes was obtained by aligning them against the NCBI non-redundant protein database and DIAMOND (version 0.9.14). On the basis of the taxonomic and functional annotation of unigenes, along with the abundance profile of unigenes, the differential analysis were carried out at each taxonomic or functional or gene-wise level by the Fisher’s exact test (non-replicated groups) or the Kruskal–Wallis test. Non-metric multidimensional scaling (NMDS) analysis was performed according to the Bray-Curtis. Alpha diversity was applied in analyzing complexity of species diversity for a sample through four indices, including Chao1, observed species, Shannon, and Simpson, and all this indices in our samples were calculated with QIIME1.

### Untargeted Metabolomic Analysis

The samples were thawed on ice, and the metabolites were extracted with 50% methanol buffer. The detailed extraction procedures were as described (Yu C.et al., 2018). The supernatant was analyzed by LC-MS to identify the metabolites. LC-MS analysis was performed on an ultraperformance liquid chromatography system (SCIEX, United Kingdom) coupled with high-resolution tandem mass spectrometer TripleTOF5600plus (SCIEX, United Kingdom). The Q-TOF was operated in both positive and negative ion modes.

The LC-MS raw data files were converted into mzXML format and then processed by the XCMS (Smith et al., 2006), CAMERA (Kuhl et al., 2012), and metaX (Wen et al., 2017) toolbox implemented with the R software (version 3.6.0). Each ion was identified by combining retention time and m/z data (LC-Bio, China). HMDB (Wishart et al., 2013) databases were used to annotate metabolites using exact molecular weight data (m/z) of the samples. Student’s *t*-test was used to detect differences in metabolite concentrations between the two groups. The *P*-value was adjusted for multiple tests using the false discovery rate (Benjamini–Hochberg). PLS-DA was used for multivariate data analysis. The metabolites with VIP > 1, *P*-value < 0.05, and fold change (FC) ≥ 1.5 or FC ≤ 0.5 were considered significantly different. In addition, significantly differential abundant metabolites were imported into MetaboAnalyst4.0 (Clarke and Haselden, 2008) database to perform pathway analysis.

### Correlation Analysis

We performed spearman correlation analysis on the differentiated metabolites screened by metabolomics, and significantly different species were obtained by metagenome sequencing analysis with *P*-value < 0.05, *r* ≥ 0.5, or *FC* ≤ −0.5. Correlation analysis was performed using the OmicStudio tools.^1^

### Statistical Analysis

Graphing and data analysis were performed using the GraphPad Prism 8.3.0 software. The *p*-value of relative abundance in the four stomach compartments at the phylum and genus levels used rank sum test to calculate. *p* < 0.05 was considered significantly different. The relative abundance partly graphics in this article were drawn using OmicStudio tools.^2^

## Data Availability Statement

The datasets presented in this study can be found in online repositories. The metagenome sequence data of four stomachs have been deposited in the Sequence Read Archive (SRA) under the accession number PRJNA77667.

## Ethics Statement

The animal study was reviewed and approved by the Committee on the Ethics of Animal Experiments at Inner Mongolia University. Written informed consent was obtained from the owners for the participation of their animals in this study.

## Author Contributions

GL, LY, and XZ designed the experiments. XZ and MG drafted the original manuscript. MY and LZ analyzed the data. DW and CB prepared the figures and tables. XW and YG collected samples. All authors contributed to discussion of results and critical revisions during the editing process.

## Conflict of Interest

The authors declare that the research was conducted in the absence of any commercial or financial relationships that could be construed as a potential conflict of interest.

## Publisher’s Note

All claims expressed in this article are solely those of the authors and do not necessarily represent those of their affiliated organizations, or those of the publisher, the editors and the reviewers. Any product that may be evaluated in this article, or claim that may be made by its manufacturer, is not guaranteed or endorsed by the publisher.

## Footnotes

1
https://www.omicstudio.cn/tool/62

2
https://www.omicstudio.cn/index
