# Supplementary material for: Comparison of Microbial Community and Metabolites in Four Stomach Compartments of Myostatin-Gene-Edited and Non-edited Cattle
Source: Front Microbiol. 2022 Mar 24;13:844962. doi: 10.3389/fmicb.2022.844962 (PMC8988179; doi:10.3389/fmicb.2022.844962)
Supplement: Supplementary file 1 [file Data_Sheet_1.docx]

Supplementary Material

## Supplementary Figures

**
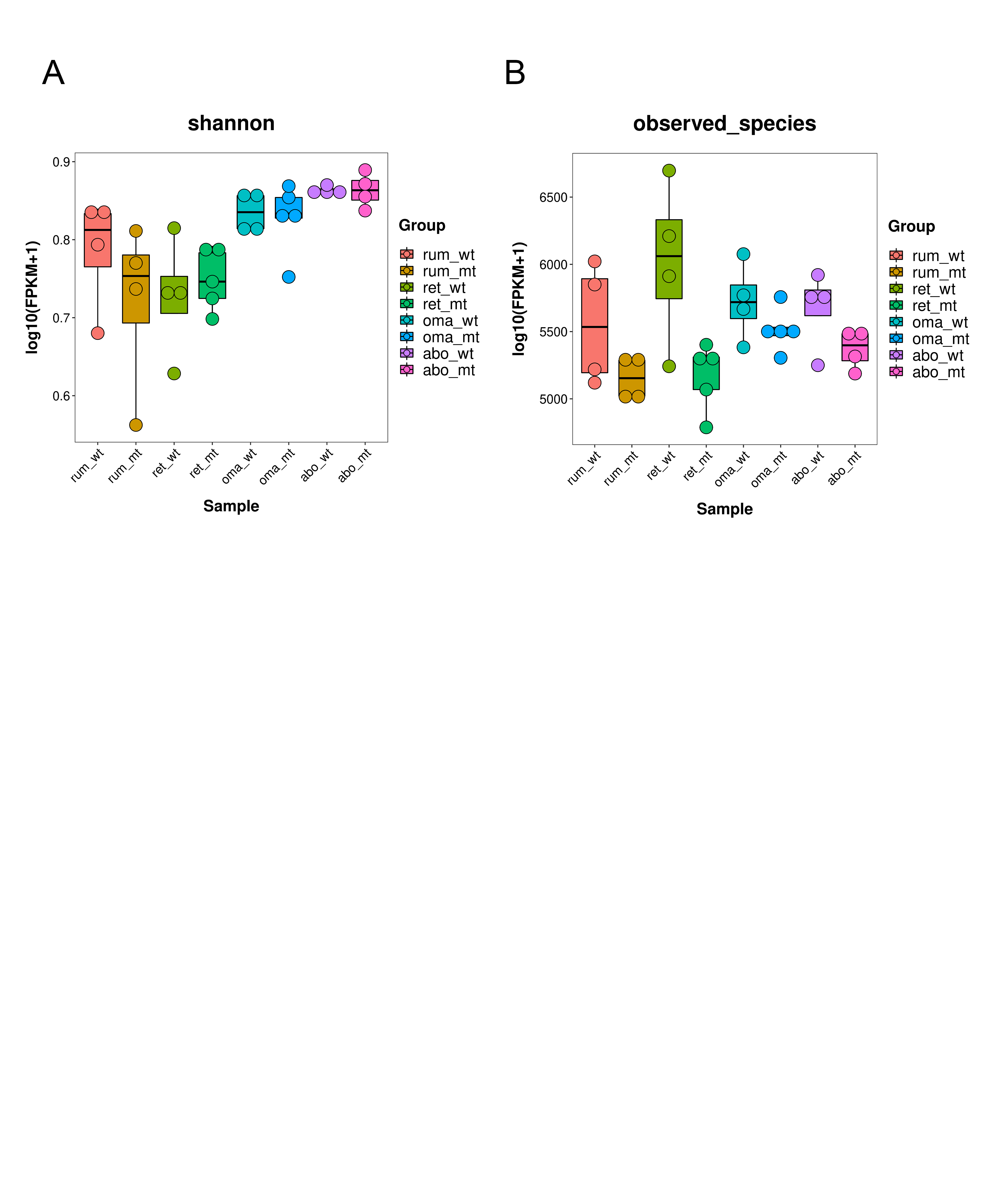
**

**Supplementary Figure 1.** The α-diversity indices of Shannon (A) and observed species (B) in both MSTN^+/−^ and WT four stomach samples. rum=rumen, ret=reticulum, oma=omasum, abo=abomasum.


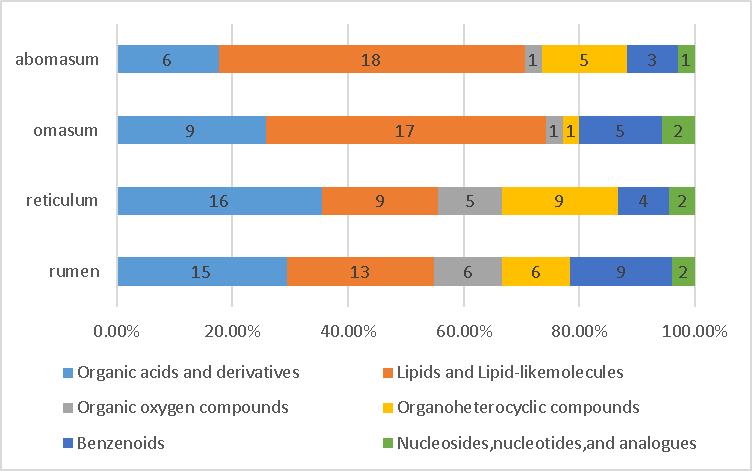


**Supplementary Figure 2.** Classification of differential metabolites according to the properties of the compounds and the proportion of each in the rumen, reticulum, omasum and abomasum.

## Supplementary Tables

**Supplementary Table 1.** Summary of sequence data generated from the four stomach samples of *MSTN^+/-^* and WT cattle.

| **Sample** | **Raw reads** | **Clean reads** | **Contigs** | **N50(bp)** |
| --- | --- | --- | --- | --- |
| rum_WT1 | 51258372 | 48351256 | 81997 | 789 |
| rum_WT2 | 47659100 | 46307724 | 80319 | 737 |
| rum_WT3 | 45391974 | 42887326 | 121096 | 788 |
| rum_WT4 | 53264890 | 52195406 | 183157 | 914 |
| rum_MT1 | 43344834 | 40579674 | 165125 | 1036 |
| rum_MT4 | 51702902 | 50464324 | 153957 | 767 |
| rum_MT3 | 35027784 | 34108822 | 123192 | 814 |
| rum_MT2 | 38515192 | 37085580 | 109328 | 873 |
| ret_WT1 | 58024010 | 56595280 | 266115 | 812 |
| ret_WT2 | 52408700 | 51254496 | 205487 | 773 |
| ret_WT3 | 55883888 | 54568082 | 235162 | 823 |
| ret_WT4 | 88687526 | 56857030 | 68458 | 877 |
| ret_MT1 | 48742298 | 45200904 | 166148 | 899 |
| ret_MT2 | 53256986 | 48411816 | 114707 | 809 |
| ret_MT3 | 51602254 | 48416124 | 93475 | 863 |
| ret_MT4 | 46776028 | 45212024 | 95908 | 753 |
| ret_MT5 | 44842306 | 43233418 | 111156 | 785 |
| oma_WT1 | 58455568 | 55563352 | 118704 | 869 |
| oma_WT2 | 41974344 | 40414650 | 90910 | 834 |
| oma_WT3 | 43222558 | 42046104 | 114012 | 795 |
| oma_WT4 | 55789098 | 52761480 | 130702 | 944 |
| oma_MT1 | 50988528 | 49707518 | 134423 | 870 |
| oma_MT2 | 45665726 | 44323160 | 134883 | 840 |
| oma_MT3 | 42470898 | 41454390 | 108806 | 819 |
| oma_MT4 | 45532500 | 44515228 | 128048 | 797 |
| oma_MT5 | 47173658 | 45307254 | 109712 | 790 |
| abo_WT1 | 55250270 | 53130130 | 54989 | 726 |
| abo_WT2 | 69691994 | 61580986 | 66207 | 789 |
| abo_WT3 | 132614206 | 128909514 | 157222 | 786 |
| abo_WT4 | 61520938 | 55444732 | 85201 | 906 |
| abo_MT1 | 43868498 | 42744606 | 108123 | 885 |
| abo_MT3 | 82720276 | 77738576 | 110528 | 868 |
| abo_MT4 | 83500782 | 80780904 | 109360 | 797 |
| abo_MT2 | 45288688 | 43538172 | 95895 | 819 |
| Total | 1872117574 | 1761690042 | 4232512 | 28246 |
| mean | 106978147.1 | 100668002.4 | 241857.8286 | 1614.057143 |
| SD | 18397821.44 | 16733705.32 | 46089.44877 | 63.69247081 |

rum=rumen, ret=reticulum, oma=omasum, abo= abomasum

**Supplementary Table 2.** The rank sum test p-value of relative abundance in the four stomach compartments at the phylum and genus level.

| **Level** | **P value** | | | |
| --- | --- | --- | --- | --- |
|  | **rumen** | **reticulum** | **omasum** | **abomasum** |
| **phylum** |  |  |  |  |
| Bacteroidetes | 0.49 | 0.19 | 0.29 | 0.49 |
| Firmicutes | 0.69 | 0.29 | 1 | 0.89 |
| Bacteria_noname | 0.49 | 0.29 | 0.41 | 1 |
| Fibrobacteres | 1 | 0.41 | 0.19 | 0.49 |
| Proteobacteria | 0.69 | 0.02^*^ | 0.73 | 1 |
| **genus** |  |  |  |  |
| Prevotella | 0.34 | 0.2 | 0.03^*^ | 0.2 |
| Bacteroides | 0.49 | 0.11 | 1 | 0.69 |
| Clostridium | 0.69 | 0.34 | 1 | 0.2 |
| Bacteria_noname | 0.49 | 0.34 | 0.41 | 1 |
| Firmicutes_noname | 0.89 | 0.2 | 0.73 | 1 |
| Fibrobacter | 1 | 0.69 | 0.19 | 0.49 |

* Stand for p<0.05 and was considered to indicate a statistically significant difference

**Supplementary Table 3.** Differential metabolites in rumen of MSTN^+/-^ and WT cattle.

| **Metabolite name** | **FC** | **p-value** | **VIP** | **MS2superclass** |
| --- | --- | --- | --- | --- |
| **Upregulated** |  |  |  |  |
| N-Acetylvaline | 2.244 | 0.017 | 1.300 | Organic acids and derivatives |
| Leucylproline | 3.158 | 0.001 | 1.451 | Organic acids and derivatives |
| Tyrosyl-Phenylalanine | 2.586 | 0.018 | 1.466 | Organic acids and derivatives |
| 3-Oxotetradecanoic acid | 3.183 | 0.007 | 1.702 | Lipids and lipid-like molecules |
| Methuyl tanshinonate | 2.558 | 0.013 | 1.621 | Lipids and lipid-like molecules |
| 4-Oxo-1-(3-pyridyl)-1-butanone | 4.389 | 0.020 | 2.096 | Organic oxygen compounds |
| 4-Deoxythreonic acid | 2.810 | 0.000 | 1.502 | Organic oxygen compounds |
| 4-(2-Aminophenyl)-2,4-dioxobutanoic acid | 4.424 | 0.009 | 2.163 | Organic oxygen compounds |
| 1H-INDOLE-3-CARBOXYLIC ACID | 4.487 | 0.014 | 2.345 | Organoheterocyclic compounds |
| Quinoline-4,8-diol | 2.368 | 0.003 | 1.242 | Organoheterocyclic compounds |
| 4-Acetyl-2(3H)-benzoxazolone | 3.040 | 0.005 | 1.148 | Organoheterocyclic compounds |
| Phenylacetic acid | 3.741 | 0.005 | 1.863 | Benzenoids |
| Mandelonitrile | 3.007 | 0.006 | 1.251 | Benzenoids |
| (+-)-JASMONIC ACID | 2.026 | 0.001 | 1.082 | Benzenoids |
| Cardanoldiene | 2.273 | 0.026 | 1.898 | Benzenoids |
| 5-Thymidylic acid | 2.086 | 0.016 | 1.073 | Nucleosides, nucleotides, and analogues |
| **Downregulated** |  |  |  |  |
| 2-Aminoisobutyric acid | 0.340 | 0.030 | 1.685 | Organic acids and derivatives |
| L-Proline | 0.326 | 0.031 | 1.666 | Organic acids and derivatives |
| Acetic acid | 0.460 | 0.011 | 1.058 | Organic acids and derivatives |
| Ureidopropionic acid | 0.322 | 0.026 | 1.570 | Organic acids and derivatives |
| L-Lysine | 0.468 | 0.033 | 1.042 | Organic acids and derivatives |
| L-Glutamic acid | 0.443 | 0.044 | 1.296 | Organic acids and derivatives |
| Citrulline | 0.278 | 0.028 | 1.954 | Organic acids and derivatives |
| N-Acetylglutamic acid | 0.411 | 0.030 | 1.723 | Organic acids and derivatives |
| N-Acetyl-L-phenylalanine | 0.432 | 0.026 | 1.154 | Organic acids and derivatives |
| 2-Hydroxyglutarate | 0.295 | 0.030 | 1.780 | Organic acids and derivatives |
| PANTOTHENATE | 0.471 | 0.045 | 1.291 | Organic acids and derivatives |
| N-Palmitoyl tyrosine | 0.336 | 0.040 | 1.353 | Organic acids and derivatives |
| D-Malic acid | 0.287 | 0.047 | 2.112 | Lipids and lipid-like molecules |
| Citramalic acid | 0.257 | 0.045 | 1.951 | Lipids and lipid-like molecules |
| Linolenelaidic acid | 0.437 | 0.046 | 1.122 | Lipids and lipid-like molecules |
| Lycopersiconol | 0.385 | 0.017 | 1.445 | Lipids and lipid-like molecules |
| 5-Hexyltetrahydro-2-furanoctanoic acid | 0.338 | 0.035 | 1.901 | Lipids and lipid-like molecules |
| Pristanoylglycine | 0.323 | 0.023 | 1.320 | Lipids and lipid-like molecules |
| Piperoic acid | 0.391 | 0.013 | 1.569 | Lipids and lipid-like molecules |
| LysoPE 12:0 | 0.460 | 0.004 | 1.757 | Lipids and lipid-like molecules |
| Beta-Glycyrrhetinic acid | 0.386 | 0.017 | 1.503 | Lipids and lipid-like molecules |
| (2xi,20beta)-2,20-Dihydroxy-3-oxo-12-ursen-28-oic acid | 0.451 | 0.042 | 1.323 | Lipids and lipid-like molecules |
| LysoPS 20:3; LysoPS 20:3 | 0.400 | 0.002 | 1.258 | Lipids and lipid-like molecules |
| D-(-)-QUINIC ACID | 0.342 | 0.044 | 2.179 | Organic oxygen compounds |
| GLUCONATE | 0.442 | 0.048 | 1.144 | Organic oxygen compounds |
| N-ACETYLNEURAMINIC ACID | 0.260 | 0.003 | 1.961 | Organic oxygen compounds |
| Guanine | 0.341 | 0.025 | 1.738 | Organoheterocyclic compounds |
| Uric acid | 0.429 | 0.040 | 1.031 | Organoheterocyclic compounds |
| SALICYLIC ACID | 0.452 | 0.025 | 1.102 | Benzenoids |
| Phenylglyoxylic acid | 0.392 | 0.048 | 1.487 | Benzenoids |
| 1-H-Inden-1-one,2,3-dihydro-3,3,5,6-tetramethyl | 0.316 | 0.032 | 1.944 | Benzenoids |
| Eudesmic acid | 0.320 | 0.049 | 1.951 | Benzenoids |
| Phenol | 0.456 | 0.024 | 1.216 | Benzenoids |
| Guanosine monophosphate | 0.164 | 0.006 | 2.669 | Nucleosides, nucleotides, and analogues |

**Supplementary Table 4. Differential metabolites in reticulum of *MSTN^+/-^* and WT cattle.**

| Metabolite name | FC | p-value | VIP | MS2superclass |  |  |
| --- | --- | --- | --- | --- | --- | --- |
| **Upregulated** |  |  |  |  |  |  |
| Acetic acid | 3.001 | 0.001 | 1.979 | Organic acids and derivatives |  |  |
| 2-Hydroxy-2-(2-oxopropyl) butanedioic acid | 2.334 | 0.009 | 1.893 | Organic acids and derivatives |  |  |
| Propyl acetate | 3.251 | 0.038 | 3.026 | Organic acids and derivatives |  |  |
| Valyl-Threonine | 3.922 | 0.001 | 2.247 | Organic acids and derivatives |  |  |
| Phenylalanyl-Methionine | 10.167 | 0.000 | 4.602 | Organic acids and derivatives |  |  |
| Isobutyric acid | 2.252 | 0.016 | 1.888 | Organic acids and derivatives |  |  |
| 2,3-Dimethyl-3-hydroxyglutaric acid | 3.649 | 0.001 | 3.014 | Lipids and lipid-like molecules |  |  |
| Dodecanedioic acid | 2.102 | 0.004 | 1.503 | Lipids and lipid-like molecules |  |  |
| (9S,10S)-9,10-dihydroxyoctadecanoate | 2.255 | 0.001 | 1.746 | Lipids and lipid-like molecules |  |  |
| Maslinic acid | 2.695 | 0.010 | 2.124 | Lipids and lipid-like molecules |  |  |
| Methyl (2E,6Z)-dodecadienoate | 2.831 | 0.000 | 2.528 | Lipids and lipid-like molecules |  |  |
| 17-Hydroxyprogesterone | 3.192 | 0.032 | 2.354 | Lipids and lipid-like molecules |  |  |
| 4-Oxo-1-(3-pyridyl)-1-butanone | 8.731 | 0.006 | 4.243 | Organic oxygen compounds |  |  |
| Allose | 10.643 | 0.013 | 4.924 | Organic oxygen compounds |  |  |
| D-(+)-TREHALOSE | 36.372 | 0.000 | 6.098 | Organic oxygen compounds |  |  |
| 4-(2-Aminophenyl)-2,4-dioxobutanoic acid | 4.662 | 0.000 | 2.741 | Organic oxygen compounds |  |  |
| 3-Hydroxypropanal | 2.156 | 0.046 | 1.357 | Organic oxygen compounds |  |  |
| 1H-INDOLE-3-CARBOXYLIC ACID | 5.604 | 0.000 | 3.223 | Organoheterocyclic compounds |  |  |
| 4-Pyridoxic acid | 2.738 | 0.006 | 2.157 | Organoheterocyclic compounds |  |  |
| 4-Acetyl-2(3H)-benzoxazolone | 2.051 | 0.000 | 1.009 | Organoheterocyclic compounds |  |  |
| Peperinic acid | 2.191 | 0.015 | 1.893 | Organoheterocyclic compounds |  |  |
| Sapidolide A | 2.284 | 0.007 | 1.659 | Organoheterocyclic compounds |  |  |
| Adenine | 2.016 | 0.049 | 1.545 | Organoheterocyclic compounds |  |  |
| 3-Methyldioxyindole | 11.227 | 0.004 | 5.128 | Organoheterocyclic compounds |  |  |
| 4-PYRIDOXATE | 2.001 | 0.003 | 1.799 | Organoheterocyclic compounds |  |  |
| 5-Hydroxyindoleacetic acid | 2.185 | 0.002 | 1.496 | Organoheterocyclic compounds |  |  |
| SALICYLIC ACID | 2.280 | 0.001 | 1.388 | Benzenoids |  |  |
| 2,4-Dihydroxybenzoic acid | 2.623 | 0.001 | 2.041 | Benzenoids |  |  |
| Prehnitene | 2.099 | 0.024 | 2.030 | Benzenoids |  |  |
| 2-Phenylacetamide | 2.028 | 0.025 | 1.177 | Benzenoids |  |  |
| Gerberinol | 15.604 | 0.025 | 5.618 | Phenylpropanoids and polyketides |  |  |
| Uridine 5'-monophosphate | 2.240 | 0.028 | 1.619 | Nucleosides, nucleotides, and analogues |  |  |
| **Downregulated** |  |  |  |  |  |  |
| Pyroglutamic acid | 0.453 | 0.012 | 1.488 | Organic acids and derivatives |  |  |
| ASPARTATE | 0.385 | 0.009 | 1.765 | Organic acids and derivatives |  |  |
| L-Glutamic acid | 0.426 | 0.008 | 1.543 | Organic acids and derivatives |  |  |
| N-lactoyl-Valine | 0.474 | 0.007 | 1.303 | Organic acids and derivatives |  |  |
| (S)-2-Azetidinecarboxylic acid | 0.357 | 0.009 | 2.122 | Organic acids and derivatives |  |  |
| THREONINE | 0.383 | 0.042 | 1.648 | Organic acids and derivatives |  |  |
| L-5-OXOPROLINE | 0.467 | 0.003 | 1.486 | Organic acids and derivatives |  |  |
| L-Proline | 0.379 | 0.021 | 1.917 | Organic acids and derivatives |  |  |
| Isoleucyl-Threonine | 0.281 | 0.013 | 1.812 | Organic acids and derivatives |  |  |
| Gamma-Glutamylleucine | 0.454 | 0.037 | 1.740 | Organic acids and derivatives |  |  |
| Triacetin | 0.457 | 0.008 | 1.702 | Lipids and lipid-like molecules |  |  |
| Ursolic acid | 0.468 | 0.047 | 1.807 | Lipids and lipid-like molecules |  |  |
| Acylcarnitine 23:0 | 0.168 | 0.012 | 3.835 | Lipids and lipid-like molecules |  |  |
| Didanosine | 0.419 | 0.014 | 2.154 | Nucleosides, nucleotides, and analogues |  |  |

**Supplementary Table 5. Differential metabolites in omasum of *MSTN^+/-^* and WT cattle.**

| **Metabolite name** | **FC** | **p-value** | **VIP** | **MS2superclass** |  |  |
| --- | --- | --- | --- | --- | --- | --- |
| **Upregulated** |  |  |  |  |  |  |
| (S)-2-Aceto-2-hydroxybutanoic acid | 2.181 | 0.002 | 1.614 | Organic acids and derivatives |  |  |
| N-Acetylvaline | 2.935 | 0.000 | 2.458 | Organic acids and derivatives |  |  |
| N-Stearoyl GABA | 2.175 | 0.010 | 1.362 | Organic acids and derivatives |  |  |
| N-Palmitoyl phenylalanine | 3.122 | 0.001 | 1.654 | Organic acids and derivatives |  |  |
| N-Palmitoyl tyrosine | 2.313 | 0.001 | 1.338 | Organic acids and derivatives |  |  |
| (Alpha-D-mannosyl)7-beta-D-mannosyl-diacetylchitobiosyl-L-asparagine, isoform A (protein) | 2.236 | 0.016 | 1.820 | Organic acids and derivatives |  |  |
| Valyl-Phenylalanine | 3.253 | 0.025 | 1.811 | Organic acids and derivatives |  |  |
| 2-Hydroxymyristic acid | 2.094 | 0.013 | 2.045 | Lipids and lipid-like molecules |  |  |
| 3-hydroxytridecanoic acid | 3.634 | 0.032 | 2.700 | Lipids and lipid-like molecules |  |  |
| Adrenic acid | 38.448 | 0.033 | 4.846 | Lipids and lipid-like molecules |  |  |
| Cinncassiol D1 | 2.317 | 0.001 | 2.062 | Lipids and lipid-like molecules |  |  |
| Nutriacholic acid | 2.213 | 0.039 | 1.504 | Lipids and lipid-like molecules |  |  |
| LysoPG 16:0; LysoPG 16:0 | 7.841 | 0.024 | 3.442 | Lipids and lipid-like molecules |  |  |
| Euscaphic acid | 2.164 | 0.011 | 1.916 | Lipids and lipid-like molecules |  |  |
| Ursolic acid | 3.094 | 0.007 | 2.887 | Lipids and lipid-like molecules |  |  |
| LysoPG 18:1; LysoPG 18:1 | 20.559 | 0.028 | 4.667 | Lipids and lipid-like molecules |  |  |
| LysoPC 14:1 | 27.179 | 0.017 | 5.615 | Lipids and lipid-like molecules |  |  |
| beta-Glycyrrhetinic acid | 2.528 | 0.003 | 2.107 | Lipids and lipid-like molecules |  |  |
| delta-Maslinic acid | 2.370 | 0.005 | 2.058 | Lipids and lipid-like molecules |  |  |
| PG 22:0; PG (3:0/19:0) | 2.269 | 0.014 | 1.399 | Lipids and lipid-like molecules |  |  |
| Acylcarnitine 16:0 | 3.038 | 0.042 | 1.986 | Lipids and lipid-like molecules |  |  |
| Acylcarnitine 23:0 | 3.063 | 0.012 | 2.527 | Lipids and lipid-like molecules |  |  |
| Phenylglyoxylic acid | 2.573 | 0.017 | 1.814 | Benzenoids |  |  |
| 3-Cresotinic acid | 2.048 | 0.001 | 1.465 | Benzenoids |  |  |
| C.I. Pigment Red 149 | 2.381 | 0.008 | 2.039 | Benzenoids |  |  |
| 4-Deoxythreonic acid | 2.127 | 0.011 | 1.560 | Organic oxygen compounds |  |  |
| Ribothymidine | 2.319 | 0.038 | 2.125 | Nucleosides, nucleotides, and analogues |  |  |
| 3-Methyl-1-butylamine | 2.456 | 0.005 | 1.509 | Organic nitrogen compounds |  |  |
| **Downregulated** |  |  |  |  |  |  |
| (R)-3-Hydroxy-5-phenylpentanoic acid | 0.454 | 0.014 | 1.481 | Organic acids and derivatives |  |  |
| GLUTAMINE | 0.080 | 0.001 | 5.201 | Organic acids and derivatives |  |  |
| Caproic acid | 0.232 | 0.034 | 3.505 | Lipids and lipid-like molecules |  |  |
| Sebacic acid | 0.491 | 0.014 | 1.373 | Lipids and lipid-like molecules |  |  |
| SALICYLIC ACID | 0.497 | 0.034 | 1.462 | Benzenoids |  |  |
| O-Desmethylnaproxen | 0.458 | 0.003 | 1.957 | Benzenoids |  |  |
| Guanosine monophosphate | 0.299 | 0.027 | 2.851 | Nucleosides, nucleotides, and analogues |  |  |
| Fentanyl | 0.306 | 0.048 | 2.760 | Organoheterocyclic compounds |  |  |
| Alpha-Hydroxy-N-desmethyltamoxifen | 0.373 | 0.020 | 2.270 | Phenylpropanoids and polyketides |  |  |

**Supplementary Table 6. Differential metabolites in abomasum of *MSTN^+/-^* and WT cattle.**

| **Metabolite name** | **FC** | **p-value** | **VIP** | **MS2superclass** |
| --- | --- | --- | --- | --- |
| **Upregulated** |  |  |  |  |
| p-Cresol sulfate | 3.035 | 0.003 | 1.812 | Organic acids and derivatives |
| PHENATURIC ACID | 2.845 | 0.014 | 2.216 | Organic acids and derivatives |
| Behenoylglycine | 2.452 | 0.022 | 1.740 | Organic acids and derivatives |
| N-Oleoyl tyrosine | 2.847 | 0.042 | 1.542 | Organic acids and derivatives |
| PE(P-16:0e/0:0) | 3.050 | 0.031 | 1.759 | Lipids and lipid-like molecules |
| LysoPE 16:0 | 2.469 | 0.001 | 1.412 | Lipids and lipid-like molecules |
| Trihydroxycoprostanoic acid | 5.005 | 0.002 | 3.495 | Lipids and lipid-like molecules |
| Delta-Maslinic acid | 5.955 | 0.001 | 3.646 | Lipids and lipid-like molecules |
| (2xi,20beta)-2,20-Dihydroxy-3-oxo-12-ursen-28-oic acid | 3.425 | 0.030 | 2.290 | Lipids and lipid-like molecules |
| Euscaphic acid | 4.474 | 0.011 | 3.516 | Lipids and lipid-like molecules |
| Beta-Glycyrrhetinic acid | 3.748 | 0.050 | 3.211 | Lipids and lipid-like molecules |
| (3beta,6alpha,19alpha)-3,6,19-Trihydroxy-12-ursen-28-oic acid | 2.941 | 0.001 | 2.411 | Lipids and lipid-like molecules |
| (2E,6E)-1-Hydroxy-2,6,10-farnesatrien-9-one | 2.223 | 0.003 | 2.085 | Lipids and lipid-like molecules |
| Alpha-Tocopherol | 2.189 | 0.006 | 1.835 | Lipids and lipid-like molecules |
| Acylcarnitine 20:0 | 5.539 | 0.050 | 5.223 | Lipids and lipid-like molecules |
| LysoPE 18:1 | 2.618 | 0.019 | 2.022 | Lipids and lipid-like molecules |
| 21beta-Hydroxyhederagenin | 2.616 | 0.001 | 2.299 | Lipids and lipid-like molecules |
| Indolelactic acid | 2.205 | 0.020 | 1.531 | Organoheterocyclic compounds |
| 1,3-Dihydro-(2H)-indol-2-one | 6.902 | 0.014 | 2.580 | Organoheterocyclic compounds |
| Mesoporphyrin IX | 2.788 | 0.005 | 2.863 | Organoheterocyclic compounds |
| 4'-Methylacetophenone | 2.159 | 0.010 | 1.982 | Benzenoids |
| C.I. Pigment Red 149 | 2.954 | 0.043 | 2.805 | Benzenoids |
| 5-Thymidylic acid | 2.197 | 0.001 | 1.821 | Nucleosides, nucleotides, and analogues |
| Acetaldehyde | 2.061 | 0.033 | 1.520 | Organic oxygen compounds |
| Alpha-Linolenoyl ethanolamide | 2.100 | 0.003 | 2.076 | Organic nitrogen compounds |
| **Downregulated** |  |  |  |  |
| L-Proline | 0.024 | 0.000 | 6.575 | Organic acids and derivatives |
| Acetic acid | 0.050 | 0.041 | 4.302 | Organic acids and derivatives |
| Caproic acid | 0.358 | 0.004 | 1.940 | Lipids and lipid-like molecules |
| 2-Methyladipic acid | 0.451 | 0.033 | 1.827 | Lipids and lipid-like molecules |
| Adrenic acid | 0.261 | 0.045 | 1.932 | Lipids and lipid-like molecules |
| LysoPC 18:3 | 0.373 | 0.037 | 1.574 | Lipids and lipid-like molecules |
| Acylcarnitine 20:4 | 0.471 | 0.046 | 2.334 | Lipids and lipid-like molecules |
| 4-Acetyl-2(3H)-benzoxazolone | 0.356 | 0.045 | 1.279 | Organoheterocyclic compounds |
| 1H-INDOLE-3-CARBOXYLIC ACID | 0.268 | 0.018 | 2.987 | Organoheterocyclic compounds |
| O-Desmethylnaproxen | 0.397 | 0.048 | 1.898 | Benzenoids |
| Coumarin | 0.266 | 0.043 | 2.516 | Phenylpropanoids and polyketides |
